# Selection and Clonal Propagation of High Artemisinin Genotypes of *Artemisia annua*

**DOI:** 10.3389/fpls.2018.00358

**Published:** 2018-03-27

**Authors:** Hazel Y. Wetzstein, Justin A. Porter, Jules Janick, Jorge F. S. Ferreira, Theophilus M. Mutui

**Affiliations:** ^1^Department of Horticulture and Landscape Architecture, Purdue University, West Lafayette, IN, United States; ^2^Department of Horticulture, University of Georgia, Athens, GA, United States; ^3^U.S. Salinity Laboratory, United States Department of Agriculture, Agricultural Research Service, Riverside, CA, United States; ^4^Department of Seed, Crop and Horticultural Sciences, University of Eldoret, Eldoret, Kenya

**Keywords:** *Artemisia annua*, artemisinin, genotypes, malaria, tissue culture

## Abstract

Artemisinin, produced in the glandular trichomes of *Artemisia annua* L. is a vital antimalarial drug effective against *Plasmodium falciparum* resistant to quinine-derived medicines. Although work has progressed on the semi-synthetic production of artemisinin, field production of *A. annua* remains the principal commercial source of the compound. Crop production of artemisia must be increased to meet the growing worldwide demand for artemisinin combination therapies (ACTs) to treat malaria. Grower artemisinin yields rely on plants generated from seeds from open-pollinated parents. Although selection has considerably increased plant artemisinin concentration in the past 15 years, seed-generated plants have highly variable artemisinin content that lowers artemisinin yield per hectare. Breeding efforts to produce improved F_1_ hybrids have been hampered by the inability to produce inbred lines due to self-incompatibility. An approach combining conventional hybridization and selection with clonal propagation of superior genotypes is proposed as a means to enhance crop yield and artemisinin production. Typical seed-propagated artemisia plants produce less than 1% (dry weight) artemisinin with yields below 25 kg/ha. Genotypes were identified producing high artemisinin levels of over 2% and possessing improved agronomic characteristics such as high leaf area and shoot biomass production. Field studies of clonally-propagated high-artemisinin plants verified enhanced plant uniformity and an estimated gross primary productivity of up to 70 kg/ha artemisinin, with a crop density of one plant m^-2^. Tissue culture and cutting protocols for the mass clonal propagation of *A. annua* were developed for shoot regeneration, rooting, acclimatization, and field cultivation. Proof of concept studies showed that both tissue culture-regenerated plants and rooted cutting performed better than plants derived from seed in terms of uniformity, yield, and consistently high artemisinin content. Use of this technology to produce plants with homogeneously-high artemisinin can help farmers markedly increase the artemisinin yield per cultivated area. This would lead to increased profit to farmers and decreased prices of ACT.

## Introduction

*Artemisia annua* L. (known a sweet Annie, annual wormwood, qinghao) is native to China and a widely naturalized and cultivated medicinal plant (Ferreira and Janick, 1997). The plant is a source of artemisinin, a sesquiterpene lactone compound that is produced in the glandular trichomes of leaves and floral parts (Duke et al., 1994; Ferreira and Janick, 1995). Artemisinin is a vital antimalarial medicine effective against drug resistant *Plasmodium falciparum.* Artemisinin combination therapies (ACTs) are recommended as a first-line treatment for drug-resistant malaria that no longer responds to quinine-derived drugs such as chloroquine or mefloquine. Globally, the World Health Organization (World Malaria Report, 2016) attributed an estimated 212 million new cases and 429,000 deaths to malaria in 2015. At the start of 2016, nearly half of the world’s population was at risk of malaria. An important additional feature is that *A. annua* compounds also exhibit antiinflammatory, antibacterial, antitumor, antiviral, and anthelmintic activities (Bhakuni et al., 2001).

Although work has progressed on the semi-synthetic production of artemisinin, field production of *A. annua* remains the principal commercial source of the compound. Satisfying the demand for artemisinin will require improved plant material containing consistently high artemisinin levels. The agricultural production of artemisia in developing countries afflicted by malaria is not only necessary, but also important to the economic well-being of farmers and their communities in these countries, where artemisia recently became a new pharmaceutical crop. Due to low and variable yield content of artemisinin the demand for artemisinin cannot be met with current plant yields (Alejos-Gonzalez et al., 2011). Artemisia growers rely on plants generated from seeds from open-pollinated plants. Thus, homogeneously high-artemisinin plants will decrease the need to expand *A. annua* cultivated land and will increase artemisinin yield per area, and possibly decrease costs of ACTs.

Although selection has considerably increased plant artemisinin concentration in the past 15 years, seed-generated plants have highly variable artemisinin content that lowers artemisinin yield per hectare. Open-pollinated cultivars produced by mass selection show variable plant-to-plant artemisinin content and biomass production due to genetic recombination. Breeding efforts to produce improved F_1_ hybrids have been hampered by self-incompatibility, which prevents conventional back-crossing to produce inbred lines. So-called hybrid cultivars based on intercrosses of two heterozygous lines still exhibit high plant-to-plant variation. For example the leading hybrid, ‘Artemis,’ exhibited extensive variation for metabolic and agronomic traits; artemisinin content on a μg/mg dry basis for individual plants ranged 22 fold, plant fresh weight varied 28 fold, and leaf area ranged 9 fold (Graham et al., 2010).

Cultivar improvement to increase artemisinin production in *A. annua* has been limited. In a global field trial of 280 distinct lines, including commercial lines and test hybrids selected for high artemisinin production, artemisinin content ranged from 0.5 to 1.4% (Larson et al., 2013). Typically, average plant artemisinin concentrations were reported to range from 0.6 to 0.7% in China^1^, and from 0.6 to 0.8% in Africa in 2013, with currently-used plants producing around 1% (Malcolm Cutler, personal communication). The bottleneck for the feasible production of artemisinin in developing countries is the lack of affordable high-quality plant material to produce consistently high artemisinin yield (Ferreira et al., 2005).

An approach combining conventional hybridization/selection with clonal propagation of superior genotypes is proposed as a means to enhance crop yield and artemisinin production. Agricultural production using improved clonal material is commonly used with many agricultural crops. Our objectives in this study were 2 fold: (1) to select *Artemisia* genotypes with high artemisinin content, and (2) to develop protocols effective for mass clonal propagation by either cuttings and/or micropropagation. Furthermore, proof-of-concept studies were conducted to assess the field performance of tissue culture-propagated plants to determine if they have consistent levels of artemisinin and acceptable agronomic characteristics in comparisons with cutting and seed-derived plants.

## Materials and Methods

### Germplasm

Seed of *A. annua* were obtained from Brazil, China, and Purdue University, and their open-pollinated progeny were grown in the greenhouse and field. Selections were made over successive generations based on agronomic characteristics such as leaf area, biomass, flowering time, and artemisinin content. Selections were cloned by cuttings and maintained in a greenhouse under long days.

### Artemisinin Chromatographic Analysis

Plant samples were oven dried at 50°C, ground to 0.5 mm particle size, extracted by refluxing in petroleum ether for 1 h, allowed to evaporate in a fume hood, then reconstituted in 20 ml of acetonitrile (two washes of 10 ml each), filtered through 0.2 μm PTFE luer-lock syringe filters and quantified (g/100 g leaf dry weight) for artemisinin, dihydroartemisinic acid, and artemisinic acid by HPLC-UV (Ferreira and Gonzalez, 2009).

### Stock Plants for Propagation Studies

Greenhouse stock plants produced from cuttings were used as a source plants for propagation studies. *A. annua* is a short-day, monocarpic plant with extremely small flowers and seeds (Wetzstein et al., 2014). To prevent flowering under fall and winter day lengths, plants were given supplemental light to maintain a 16-h photoperiod. Plants were maintained in pots (19 cm diameter, 3.8 L) containing Fafard 3B medium (Conrad Fafard, Agawam, MA, United States) in a glass greenhouse set at 25°C. Propagation studies were performed before and concurrently with selections of elite germplasm, and included studies using a Brazilian genotype (3M, CPQBA) and field-selected clones (B4, B6, C1, C10, MP11, P63, P137) derived from crosses. All clones were selected for high-artemisinin concentration and biomass production.

### Propagation by Cuttings

Various types of cuttings (terminal, lateral, one-node, and two-node) were obtained from greenhouse-grown clones of B4 and C10 clones. Preliminary rooting studies indicated that a 1500 ppm indole-3-butyric acid, potassium salt (KIBA) dip was effective for root development. Cuttings were dipped 5 s and inserted in growing media under mist. Rooting was evaluated after 14 days.

### Initiation and Establishment of Aseptic Cultures

A range of explants types (shoot tips, leaves, nodes, floral bud, and seedling parts) were evaluated in preliminary experiments, and a series of different sterilization combinations were assessed. High numbers of clean, regenerable cultures were obtained with shoot tip explants using the following surface sterilization and culture initiation methods. Shoot tips (1 to 1.5 cm long) with young unexpanded leaves were collected from stock plants grown in the greenhouses. The basal, older leaves were removed, retaining leaves ≤ 0.5 cm long. Explants were washed for 30 min in tap water containing a two drops of antibacterial hand soap (SoftCIDE^®^, VWR, Suwanee, GA, United States), and then rinsed in water for 15 min. This was followed by sequential immersion in 70% ethanol for 20 s, 1.2% sodium hypochlorite containing 1–2 drops of Tween-20 surfactant for 10 min with agitation, and three rinses in sterile distilled water for 5 min each. After surface sterilization, shoot tips were placed on shoot induction medium consisting of Murashige and Skoog (MS) macro and micro salts (Murashige and Skoog, 1962), B5 vitamins (Gamborg et al., 1968), 0.1 mg L^-1^ myo-inositol, 0.2 mg^-l^ L 6-benzylaminopurine (BA), 0.05 mg L^-1^ kinetin (Kin), 30 g L^-1^ sucrose, and 4 g L^-1^ Gel-Gro (ICN Biochemicals, Aurora, OH, United States). The medium was adjusted to pH 6.0, dispensed in 20 mL aliquots into test tubes, and autoclaved at 121°C for 20 min. Cultures were maintained under a 16/8-h (light/dark) photoperiod under cool-white fluorescent lights (Osram Sylvania, Mississauga, ON, Canada) with 70 μmol m^2^ s^-1^ irradiance at 25 ± 10°C. Cultures were transferred to fresh medium every 3 weeks and maintained in glass baby food jars (66 mm × 59 mm). These primary shoot cultures served as a source of explants for subsequent medium optimization studies for shoot proliferation and rooting.

### *In Vitro* Shoot Regeneration Studies

Plant growth regulator screenings for shoot regeneration were conducted using material from stock cultures of the C10 clone. Small shoot clumps (1 cm × 1 cm) were inoculated on media containing different concentrations of BA (0, 0.89, 2.22, 4.44, and 8.88 μM) and naphthaleneacetic acid (NAA) (0, 0.27, 0.54 μM) to assess shoot proliferation and regeneration efficacy. The components of the media were the same as for culture initiation, except that plant growth regulators were modified. The media were dispensed into glass baby food jars with 30 ml of medium per jar. Treatments were replicated using 24 jars per medium type. Tissues were subcultured to fresh medium at 3 weeks. Shoot and callus were separated and fresh weights were recorded for plants from all jars after 6 weeks; tissues were oven dried to determine dry weights. Nine cultures were randomly selected to determine the average number of shoots per jar that were taller than 0.6 cm. Based on results of screening studies, further refinement studies were conducted to evaluate the effect of plant growth regulators on shoot and callus production. Shoot clumps (1 cm × 1 cm) from stock cultures of the 3M genotype were placed on media with different concentrations of BA (0, 0.89, 1.79, 2.67, or 3.56 μM) and NAA (0 or 0.27 μM). Except for the plant growth regulators, the components of the media, culture vessel, and growth conditions were the same as in preliminary screening studies. Twenty four jars per medium type were used for shoot and callus growth assessments; with nine jars were used for counting total numbers of shoots. The response of four different genotypes was evaluated using shoots initiated from 3M, C10, B6, and MP11. Shoot clumps (1 cm × 1 cm) were placed on media with BA (0.89, 1.79, or 3.56 μM) and NAA (0.27 μM) with 72 replicates for each plant growth regulator combination. Fresh weight and dry weight for both callus and shoots were determined after 6 weeks. A subset of 24 jars per treatment was used to determine the number of shoots per culture.

### *In Vitro* Rooting

To evaluate rooting, shoots from stock cultures of clones C10 and MP11 genotypes were placed on MS medium with B5 vitamins (Gamborg et al., 1968), 0.1 mg L^-1^ myo-inositol, 30 g L^-1^ sucrose, and 4 g L^-1^ Gel-Gro (ICN Biochemicals, Aurora, OH, United States), supplemented with different concentrations of indole-3-butyric acid (IBA) (0, 2.4, 4.9, and 9.8 μM). Percent rooting, number of roots, and number of lateral roots were assessed after 4 weeks. Studies indicated that better quality shoots were obtained following a shoot elongation step when shoots were subcultured into Magenta boxes containing basal medium for 1 week prior to transfer onto rooting medium. The rooting performance of elongated shoots from several genotypes was assessed on rooting medium containing 9.8 μM IBA.

### Field Performance of Tissue Culture-Derived Plants

The performance of plantlets derived from tissue culture was evaluated in field studies conducted in Athens, GA. Corresponding plant material from two genotypes, 3M and MP11, were propagated either via tissue culture or by cuttings and planted in field plots to compare the effect of propagation method on artemisinin concentration. In addition, seedling plants derived from open-pollinated seed collected from UGA research plots were transplanted into test plots to compare the performance and variability of vegetatively-propagated versus seed-produced plants. For the production of rooted cuttings, the base of shoot tip cuttings 10 cm tall from greenhouse stock plants were dipped into an aqueous solution of 1500 ppm KIBA for 5 s, planted in 72-cell plug trays in commercial potting mix (Fafard 3B; Conrad Fafard, Agawam, MA, United States), and covered with clear plastic propagation domes (Humi-dome; Hummert, Earth City, MO, United States). Leaf samples from four plants of each propagation type were collected for artemisinin analysis as described below. Propagules were planted in the field on June 24 and harvested September 10. To compare plant growth characteristics, the total biomass of the MP11 genotype was determined using six plants per propagation method. At harvest, five leaves and stems were manually separated into component parts, and oven dried to determine plant dry weight.

### Effect of Growing Conditions on Sesquiterpenes

Leaf concentrations of artemisinin, artemisinic acid, and dihydroartemisinic acid were quantified by HPLC-UV (Ferreira and Gonzalez, 2009) from clones grown under greenhouse, field, or tissue culture conditions. If artemisinin concentration can be accurately assessed from greenhouse-grown plants, this would streamline selection in that field screenings would not be necessary. Comparisons were made using three genotypes: 3M, MP11, and C10. Greenhouse plants were grown and maintained in pots as described under plant material. Tissue culture plants were regenerated from shoots collected from stock shoot cultures. Field material was from plants propagated by cuttings and grown in research plots.

## Results

### Selection for Improved Genotypes

**FIGURE 1:**
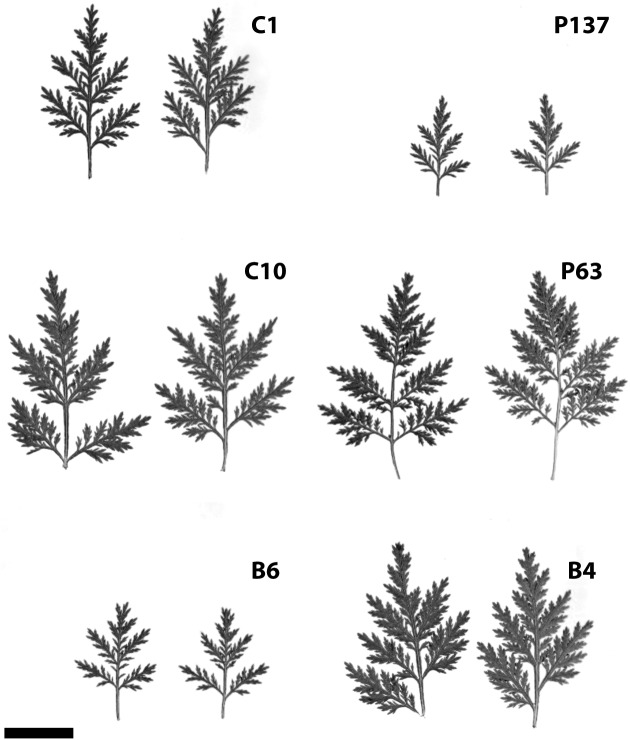
**(A–F)** Variation in leaf size of six selected clones of *Artemisia annua* grown under field conditions (bar = 2 cm).

Hybridization and selection studies identified a number of excellent genotypes in terms of both artemisinin content and agronomic characteristics. Data from field trials of six promising genotypes are shown in **Table 1**. Artemisinin leaf concentration for the six genotypes (C1, C10, B6, P137, P63, and B4) had 2-year field averages ranging from 1.6 to 2.16%. Significant differences in plant height, plant width, and stem dry weight were observed. Two genotypes (P63 and B4) exhibited a smaller stature than the others, but size differences did not necessarily relate to artemisinin plant production. The six genotypes had marked differences in leaf size and morphology (**Figure 1**). In addition to artemisinin concentration, plant size, and leaf area are important factors as they directly influence total artemisinin production. The genotype with the highest artemisinin content, C1, had an average artemisinin content of 2.16% and, based on dry leaf biomass and crop density of 1.0 plant/m^2^, had an estimated gross primary productivity of 69.6 kg of artemisinin/ha. Considering a commercial extraction efficiency of 75%, C1 would produce 52.2 kg of artemisinin/ha. P137 was the second highest yielding genotype due to its high leaf dry weight production. The six selections, particularly C1, C10, B6 and P137, represent substantial improvements in artemisinin production compared to current commercial plantings using seed of open-pollinated plants that typically produce approximately 1% artemisinin or less.

**Table 1:** Artemisinin concentration per plant, and kg/ha from six selected clones of *Artemisia annua*.

Genotype	Artemisinin (%)			Plant height (cm)^z^	Plant width (cm)^z^	Stem dry weight (kg)^z^	Leaf dry weight^z^		Artemisinin (kg/ha)^z^
	2012	2013	Average				(kg)	(t/ha)	
C1	2.13	2.19	2.16a	179a^y^	113a	0.69a	0.32ab	3.22	70.6
C10	1.93	2.13	2.03ab	188a	106ab	0.77a	0.28ab	2.81	59.9
B6	1.65	2.21	1.93ab	181a	112ab	0.67a	0.26ab	2.61	57.8
P137	1.93	1.68	1.81bc	192a	111ab	0.81a	0.39a	3.88	65.2
P63	1.81	1.53	1.67c	144b	100bc	0.44b	0.25b	2.46	37.7
B4	1.83	1.37	1.60c	140b	89c	0.34b	0.27ab	2.65	36.3

*^z^2012 data. ^y^Mean separation Tukey test, α = 0.05. Artemisinin productivity is based on leaf dry weight, 1 plant/m^2^, and without considering a commercial extraction loss of 25%. Means within the same column followed by the same letter are not significantly different.*

### Propagation by Cuttings

Vegetative propagation methods were evaluated in order to assess if methods for mass clonal propagation could be developed. Stem cuttings were initiated using tip and nodal tissues given KIBA treatments to assess rooting. There were clonal differences observed with clone B4 rooting better than C1. Nonetheless, all cutting types rooted well with 74% rooting or higher obtained (**Table 2**). These results indicate that both tip and nodal cuttings can be rooted with an IBA-hormone dip combined with misting is a very effective way to mass propagate selected *A. annua* clones.

**Table 2:** Effect of cutting type on rooting of two genotypes of *Artemisia annua*; *n* = 6 replicates of 18 cuttings each.

	B4 clone		C1 clone	
Cutting type	Rooting (%)^z^	Root dry weight (mg)	Rooting (mg)	Root dry weight (mg)
Tip	97.2 a	4.0 ab	74.1 a	11.3 a
Lateral tip	89.8 b	6.0 a	88.3 a	8.9 ab
One node	100.0 a	3.2 b	87.4 a	6.2 ab
Two nodes	98.2 a	4.7 ab	90.5 a	3.4 b
Average	96.3	4.3	85.1	7.4

*^z^Mean separation Tukey test, α = 0.05. Means within the same column followed by the same letter are not significantly different.*

### *In Vitro* Shoot Regeneration

**FIGURE 2:**
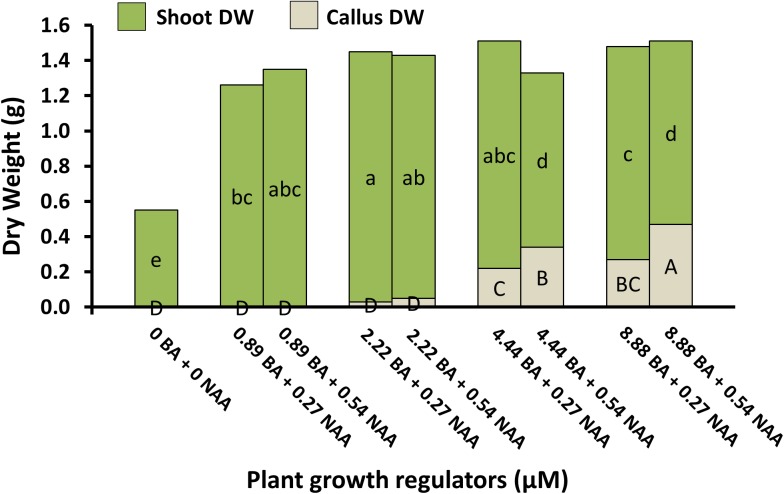
Shoot and callus dry weights of *A. annua* genotype C10 shoot cultures under different plant growth regulator combinations after 6 weeks. Means separated within shoot dry weight (lower) or callus dry weight (upper) by LSD, *p* = 0.05. Treatment means (*n* = 24) with the same letter within a group are not significantly different.

Micropropagation was also explored for *A. annua*. In preliminary experiments, several explant types were evaluated including shoot tips, nodes, leaves, and parts from axenically-germinated seedling-regeneration experiments. Shoot tips proved to be extremely regenerable when initiated on media with BA, and had relatively low contamination rates (0 to 9%) using the protocols described. Depending on genotype, 68 to 89% of explants responded on initiation medium producing shoot cultures that were used as stock cultures for shoot regeneration studies. Leaves and nodes did not perform as well (data not shown). Highest shoot growth was achieved with 2.22 BA + 0.27 μM NAA (**Figure 2**). Higher concentrations of BA and NAA resulted in undesirable increased callus production. A subsequent refinement study evaluated BA concentrations (**Table 3**). Although the greatest shoot fresh and dry weights were achieved with 1.78 μM BA + 0.27 NAA, significantly higher shoot numbers (1.5 times greater) were obtained using a lower BA concentration (0.89 μM BA). In contrast, the two highest BA levels evaluated were ineffective and produced short, chlorotic and browning shoot clumps. To assess the widespread applicability of the shoot regeneration methods, shoot and callus growth were evaluated for five *A. annua* genotypes (**Table 4**). The results over all genotypes confirmed that high numbers of shoots can be produced using BA in combination with NAA; excessive callus growth occurs with high BA concentrations. Furthermore, different genotypes exhibited variable responses in shoot and callus growth. For example, shoot numbers exhibited a 3.5 fold difference in genotype C10 versus 3M. This indicates that optimization on an individual genotype basis may be warranted.

**Table 3:** Effects of plant growth regulators on shoot and callus production in *Artemisia annua* cultures after 6 weeks; *n* = 24.

BA (μM)	NAA (μM)	Shoot		Callus		No. shoots^y^
		FW (g)	DW (g)	FW (g)	DW (g)	
0	0	0.69cz	0.13c	0b	0b	1.4c
0.89	0.27	8.88 b	0.86 b	1.83 a	0.20 a	42.3 a
1.78	0.27	12.56 a	1.08 a	1.30 ab	0.13 ab	26.9 b
2.67	0.27	9.42 b	0.92 ab	2.45 a	0.25 a	20.1 b
3.56	0.27	9.88 b	0.81 b	2.45 a	0.24 a	12.2 bc

*^z^Mean separation by LSD, p = 0.05. Means within the same column followed by the same letter are not significantly different. ^y^n = 9.*

**Table 4:** Effects of BA in combination with NAA on shoot and callus growth for different genotypes of *Artemisia annua* after 6 weeks.

Treatment (μM)		FW (g)		DW (g)		WC (%)		No. shoots^y^
BA	NAA	Callus	Shoot	Callus	Shoot	Callus	Shoot	
0.89	0.27	1.2 cz	14.4 b	0.1 c	1.3 a	1.1 c	13.2 b	55.2 a
1.78	0.27	2.5 b	15.6 ab	0.2 b	1.3 a	2.3 b	14.2 ab	64.4 a
3.56	0.27	3.9 a	17.3 a	0.3 a	1.4 a	3.5 a	15.9 a	54.1 a
**Genotype**								
C10		0.8 c	18.5 b	0.09 c	1.6 a	0.7 c	16.8 b	91.4 a
B6		5.0 a	16.0 c	0.38 a	1.4 b	4.6 a	14.6 c	59.2 b
MP11		1.9 bc	21.9 a	0.16 bc	1.7 a	1.7 bc	20.2 a	53.8 b
3M		2.4 b	6.6 d	0.23 b	0.6 c	2.1 b	6.0 d	26.0 c

*^z^Mean separation by LSD, p = 0.05. Means within the same column followed by the same letter are not significantly different. ^y^n = 24 cultures.*

### *In Vitro* Rooting

Shoots placed on rooting medium containing IBA readily produced roots within 4 weeks (**Table 5**). Percent rooting was significantly higher at the two higher IBA concentrations evaluated with 100% rooting obtained for C10 and 92% for MP11 genotype. While IBA at 9.8 μM produced significantly higher numbers of primary and lateral roots than other concentrations, there was continuing shoot proliferation and callusing on the base of shoots. An intermediate treatment was used to overcome this problem where shoots were placed on basal medium with no plant growth regulators to promote shoot elongation and arrest continued shoot proliferation. The rooting responses of elongated shoots obtained from four different genotypes were assessed using a rooting medium supplemented with 9.8 μM IBA (**Table 6**). Percent rooting with 3M, C10, and B6 genotypes was greater than 98%. Significantly lower rooting at 78% was obtained with MP11. Root number was significantly affected by genotype, ranging from 6 to 17 roots per shoot.

**Table 5:** Effects of IBA concentration on the rooting of regenerated shoots in two genotypes of *Artemisia annua* after 4 weeks.

IBA (μM)	C10 clone			MP11 clone		
	Rooting (%)	No. roots	No. lateral roots	Rooting (%)	No. roots	No. lateral roots
0	8.3 cz	0.1 c	0.3 b	22.2 c	1.2 c	1.0 b
2.4	66.7 b	2.2 c	0.9 b	70.4 b	4.9 c	1.3 b
4.9	83.3 ab	5.8 b	2.3 b	92.6 a	12.3 b	3.7 b
9.8	100.0 a	13.6 a	5.1 a	92.6 a	26.3 a	8.6 a

*^z^Mean separation by LSD, p = 0.05. Means within the same column followed by the same letter are not significantly different.*

**Table 6:** Rooting response of different *Artemisia annua* genotypes after 4 weeks on media containing 9.8 μm IBA; *n* = 50.

Genotype	Rooting (%)	No. roots
3M	98 az	17.0 a
C10	100 a	14.5 ab
B6	100 a	9.6 bc
MP11	78 b	6.2 c

*^z^Mean separation by LSD, p = 0.05. Means within the same column followed by the same letter are not significantly different.*

### Micropropagation Protocol

**FIGURE 3:**
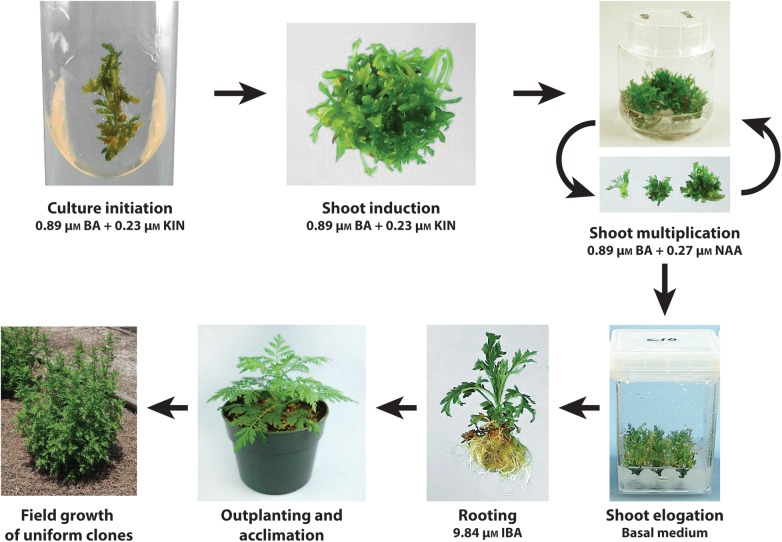
Scheme showing the tissue culture protocol developed for the clonal propagation of *A. annua*. Cultures can be initiated from shoot tip or leaf explants of established plants placed on a shoot induction medium. Shoot multiplication is facilitated on a medium with BA and KIN. Transfer of shoots to a basal medium promotes the elongation of shoots, prevents continued shoot proliferation and enhances rooting of shoots when transferred to a medium with IBA. Following acclimation, plantlets outplanted into the field exhibit uniform growth.

A tissue culture protocol via shoot proliferation was successfully developed for *A. annua*. A scheme depicting culture initiation, shoot induction, shoot multiplication, shoot elongation, rooting, and outplanting into the field is shown in **Figure 3**. Cultures can be initiated from shoot tip or leaf explants of established plants. Unlike systems requiring seedling explants, clonal lines thus can be established from genotypes identified as having high artemisinin leaf concentration and excellent agronomic characteristics.

### Field Performance of Tissue Culture-Derived Plants

Field studies evaluated the performance of two clonally propagated genotypes and of plants derived from seed. Plants propagated both by tissue culture and cuttings produced similar artemisinin (**Table 7**). The relative standard deviation (RSD) values varied from 5.86 to 14.7%. In contrast, plants generated from open-pollinated seedlings had an RSD of 33.65% (**Table 7**), indicating that the variance in artemisinin content was higher with the seedling population. Clonal propagation, whether by tissue culture or cuttings, produced greater uniformity than that obtained from seedlings regarding artemisinin concentration.

**Table 7:** Artemisinin concentration (g/100 g DW) of leaves from field-grown plants of two cloned genotypes (3M and MP11) propagated by tissue culture or rooted cuttings.

Plant material	Propagation method	Artemisinin (%) (*n* = 4)	RSD^z^ (%)	Significance (*p*)
3M clone	Tissue culture	0.78	14.4	0.83^ns^
	Cutting	0.76	14.7	
MP11 clone	Tissue culture	0.79	10.4	1.00^ns^
	Cutting	0.78	5.86	
Open pollinated seedlings		0.70	33.65	N/A

*Variability (RSD) was compared to plants derived from open-pollinated seed. Plants were placed in the field on 24 June and harvested on 14 September. ^z^RSD, relative standard deviation; ns, no significant difference.*

Plant growth characteristics of field-grown plants propagated using cuttings or tissue culture are shown in **Table 8**. No significant differences were observed in leaf dry weight, shoot dry weight, root dry weight, total plant dry weight, leaf:shoot ratio, or leaf area. This indicates that plant growth characteristics of tissue-cultured plants are similar to that of conventionally propagated cuttings.

**Table 8:** Plant growth characteristics of field-grown plants propagated by tissue culture or cuttings.

Propagation method^z^	Dry weight (g)				Leaf:shoot ratio	Leaf area (cm^2^)
	Leaf	Shoot	Root	Total plant		
Cuttings	225a^y^	446a	124a	796a	0.52a	12.8a
Tissue culture	198a	415a	102a	714a	0.48a	13.1a

*^z^n = 6 plants per propagation method. Planting date 24 June; harvest date 14 September. ^y^Mean separation by LSD, p = 0.05. Means within the same column followed by the same letter are not significantly different.*

### Effect of Growing Conditions on Sesquiterpenes

Artemisinin, dihydroartemisinic acid, and artemisinic acid were evaluated from leaves obtained from plants grown under field, greenhouse, or tissue culture conditions. Plants grown under field and greenhouse conditions produced very similar levels of artemisinin (**Table 9**). Clone 3M had average artemisinin concentrations of 1.80 and 1.94% under greenhouse and field conditions, respectively, while the MP11 genotype had respective concentrations of 1.25 and 1.21% (**Table 9**). The concentrations of dihydroartemisinic acid and artemisinic acid in those plants were also very similar under both greenhouse and field conditions. However, artemisinin concentration in tissue culture plants were 40–97 times less than greenhouse or field grown plants (**Table 9**).

**Table 9:** The effect of growing conditions on artemisinin (ART), dihydroartemisinic acid (DHAA), and artemisinic acid (AA) content in three genotypes of *Artemisia annua*.

Genotype^z^	Growth conditions	ART (%)	DHAA (%)	AA (%)
3M	Field	1.94 ± 0.23	0.80 ± 0.13	0.10 ± 0.02
	Greenhouse	1.80 ± 0.21	0.68 ± 0.12	0.07 ± 0.01
	Tissue culture	0.02 ± 0.02	0.02 ± 0.01	0.00 ± 0.00
MP11	Field	1.21 ± 0.07	0.53 ± 0.16	0.07 ± 0.01
	Greenhouse	1.25 ± 0.07	0.44 ± 0.04	0.06 ± 0.006
	Tissue culture	0.02 ± 0.06	0.08 ± 0.032	0.01 ± 0.002
C10^y^	Greenhouse	1.23 ± 0.06	1.24 ± 0.12	0.32 ± 0.02
	Tissue culture	0.03 ± 0.01	0.10 ± 0.05	0.01 ± 0.002

*^z^n = 4 for each genotype and growing condition. ^y^Field material unavailable at the time of analysis.*

These results confirm that greenhouse-grown plants can be used for selection and biochemical analysis of artemisinin and related compounds, and provide accurate estimations of plant performance in the field. Current studies concur with the effectiveness of using greenhouse grown material for screening artemisinin content as was used by Graham et al. (2010).

## Discussion

The present study describes several selections we have identified that produce high levels of artemisinin (up to 2.16%) and which exhibit superior agronomic characteristics including high leaf area and biomass production. When grown as clonal plots, artemisinin yields per hectare were remarkable. Our best genotype, C1, when planted at the density of 1 plant/m^2^, had a 2.16% artemisinin content and produced 3.22 t/ha leaf dry weight, for an estimated gross primary productivity of 69.6 kg of artemisinin/ha. In nature, artemisinin content in the leaves and flowers of wild-type *A. annua* is low (0.03–0.8%) (Charles et al., 1990; Ferreira et al., 1995; Alejos-Gonzalez et al., 2011; Liu et al., 2011). Improving artemisinin content through genetic breeding has increased content. Nonetheless, yields are usually 0.5–1.2% artemisinin, yield of dry leaf per hectare varies from 1.5 to 2 t per hectare and reports indicate that a yield of 6–14 kg of artemisinin per hectare from well-managed plantations can be expected (CNAP, 2017).

Growers in Madagascar have used cuttings of selected plants for their commercial crops for their desirable agronomic characteristics. Although this practice is labor-intense it produces more robust plants than the ones generated from seedlings (Ellman and Bartlett, 2010). These authors also mentioned a plant density of 1 plant/m^2^ for robust plants and up to 3 plants/m^2^ for less robust plants. However, there is no mention of cuttings being used to produce a crop with robust plants that are also higher in artemisinin concentration or for a homogeneous crop regarding artemisinin concentration per plant.

Artemisinin concentration is an extremely variable trait in *A. annua* ranging from 0.5 to 1.07% (Delabays et al., 2001). Although the high-heritability of artemisinin content has been experimentally confirmed by broad and narrow-sense heritability (Ferreira et al., 1995; Delabays et al., 2002), production of F_1_ hybrids produced from homozygous inbred plants has been problematic because *A. annua* exhibits self-incompatibility (Peter-Blanc, 1992; Wetzstein et al., 2014) which results in the inability to produce homozygous lines by inbreeding. The so-called hybrid seed presently available for *A. annua* is produced by crossing two heterozygous and genetically-different parental genotypes, which results in highly variable progeny. The production of inbred lines can potentially be accomplished by producing double haploids (Liu et al., 2016), but this technique must be demonstrated for *A. annua* and would be an extensive effort.

Vegetative propagation and the use of clones are standard methods used in horticultural crop production of floriculture, vegetable and major plantation crops. Cloning produces plants that are genetically identical, maintain desired characteristics, and is a means for the immediate capture of improvements in species that are difficult to breed by conventional means such as *A. annua*.

Methods for the vegetative propagation of clonal lines selected for high artemisinin have been demonstrated in this paper using two strategies: (1) *in vitro* tissue culture and (2) cutting propagation. Further, proof of concept studies verified that clonally-propagated plants provide consistent sesquiterpene production originally found in mother plants, and crop uniformity. Plants were morphologically similar in vegetative characters such as branching, shoot growth patterns, and leaf morphology. Observations in our clonal plots indicated that time-to-flowering was consistent within a genotype (data not presented) which will aid in time-to-harvest decisions. Harvest is often timed for the peak in artemisinin and biomass, which were prior to flowering for both early and late flowering clones (Delabays et al., 2001; Ferreira, 2008) making reproductive uniformity within a field plot an advantage.

Micropropagation or tissue culture affords some distinct advantages as a propagation strategy including the ability to produce millions of contaminant-free elite plants. High propagation rates were achieved in the current study using adventitious shoot formation. The methods were effective for many genotypes, and cultures lines can be initiated from mature greenhouse or field-grown plants. Unlike culture systems that require axenically-germinated seed or young seedling tissues for culture initiation, plants with proven artemisinin content and field performance can be used. Some genotypic differences in culture were observed. Thus, media optimization studies are anticipated to be necessary for different genotypes. Also, tissue culture plants analyzed by HPLC-UV, which had no developed roots, and had only traces or no artemisinin, dihydroartemisinic acid, or artemisinic acid. These results agree with a previous report that rootless *A. annua* plants had negligible amounts of artemisinin when compared to control rooted tissue culture plants (Ferreira and Janick, 1996).

Although plant tissue culture has been used to produce natural and pharmaceutical products in a number of systems, the current study confirms that shoot cultures are a poor source for sesquiterpenes. The production of artemisinin by means of cell, tissue, or organ cultures is not viable (Nair et al., 1986; Ferreira and Janick, 1996). The low artemisinin concentrations of *A. annua* leaves developed *in vitro* is a common observation (Covello et al., 2007), and may be a function of the scarcity of glandular trichomes in tissue-cultured shoots (H. Wetzstein, personal communication). Callus grown in tissue culture contains little or no artemisinin (Nair et al., 1986; Ferreira and Janick, 1996). Production of artemisinin in bioreactors has not achieved high artemisinin levels (Kim et al., 2001; Liu et al., 2003, 2006). Although nutrients, growth regulators, oxygen, and culture systems play important roles in optimizing final artemisinin concentration (Weathers et al., 1997; Kim et al., 2001; Mohammad et al., 2014), bioreactors are not a commercially feasible approach to produced artemisinin. An approach using genetically engineered yeast cultures has produced artemisinic acid (Ro et al., 2006), but commercial production of artemisinin using this technology has not materialized. More recently, artemisinin has been produced in engineered moss (Khairul Ikram et al., 2017), but with a very low concentration (0.021%). Thus, we envision the use of *in vitro* culture in *A. annua* as a method for micropropagation.

Propagation by cuttings was also found to be an effective method for propagation of *A. annua.* High rates of rooting were obtained using both tip and nodal cuttings. Plant material could be provided to growers as rooted cuttings or as unrooted cuttings that would be placed in misted field nurseries for rooting before field transplanting. Use of cuttings is widespread for ornamentals production and about 5 billion cuttings are produced per year in tropical countries and air-freighted to temperate growers (Faust et al., 2017). Cuttage operations are simpler and require less infrastructure and expertise than needed for tissue culture. However in this case, special greenhouse facilities would be required for cuttage technology because artemisia is a short-day, monocarpic species which flowers, sets seed, and then dies if plants are not maintained under long days. Mother plants must be maintained in greenhouses supplied with artificial light to remain vegetative.

Generally, vegetative propagation is more costly (per unit propagule) than seed propagation (Hartman et al., 2011). Facilities needed for vegetative propagation include protected culture, mist/high humidity for cuttings, and laboratories if tissue culture is employed (autoclaves, aseptic hoods, and growth rooms). However for many crop species, the superiority, consistent quality, and uniformity of clonal plants justifies the higher propagation costs. An economic analysis would have to be made to determine if the increase in yield obtained from high-artemisinin clones would compensate for the increased cost of planting stock. Currently, cultivation of *A. annua* is accomplished by seed. Because seed are so small (10–15,000 per gram), sowing and germination of seed in a nursery, with transplantation to allow seedlings to make additional growth prior to transplanting in the field are commonly practiced, particularly when purchased high-value seed is used (Laughlin et al., 2002). Thus, some of the costs of clonal propagation, i.e., shoot elongation and explant rooting, may compare similarly to current steps in seed production, such as thinning and transplanting. A high-efficiency clonal micropropagation system could be an economically-feasible alternative to current seed propagation practices if regeneration rates are high and enhanced artemisinin production is sufficiently elevated.

Commercial micropropagation is often limited to crops generating high unit prices, including ornamental plants and food crops (Kane et al., 2015). The application of additional *in vitro* systems such as somatic embryogenesis, bioreactors, and temporary immersion systems for plant propagation could provide extremely high regeneration rates, are amenable to scale up, can provide higher quality plant material, and thus can significantly decrease the cost of tissue-culture derived plants (Etienne and Berthouly, 2002; Georgiev et al., 2014). In horticultural crop production, tissue culture is often used to maintain and multiply disease-free mother plants used for cutting propagation. Clones of high-artemisinin lines could be propagated in individual farms or as a separate operation which is common in many clonally-propagated crops. Cultures can be maintained and shipped to locations for rooting and to set up plant blocks from which mother plants are propagated for cuttings.

The goals of these studies were to select superior high-artemisinin-producing genotypes of *A. annua*, and to develop methodology to propagate these superior genotypes. The approach of combining conventional hybridization/selection of superior genotypes with clonal propagation is a means to enhance crop yield and artemisinin production. The clonal propagation of superior high artemisinin-yielding cultivars would provide significant improvements in crop production, particularly since to date even the best seed available produce plants highly variable in artemisinin content and agronomic characteristics (Graham et al., 2010). Proof of concept studies substantiated that both tissue culture-regenerated plants and those produced by cuttings performed better than plants derived from seed in terms of uniformity, yield, and consistently high artemisinin content. Using vegetative propagation to produce plants with homogeneously-high artemisinin can provide a consistent source of improved plant material that could rapidly become available to local farmers, help growers to markedly increase artemisinin yield per cultivated area, and feasibly be adopted in the world’s major production areas of Southeast Asia and Africa. Cost-benefit analysis is needed to reveal best management practices employing sustainable and profitable production criteria.

## Conclusion

The results of these studies indicate that selection can produce plants of *A. annua* with artemisinin levels above 2%. The current study identified four clones with artemisinin levels ranging from 1.8 to 2.2% and possessing improved agronomic characteristics such as high leaf area and shoot biomass production. Artemisinin production from these genotypes produced an estimated gross primary productivity from 58 to 70 kg/ha artemisinin, with a crop density of 1 plant m^-2^. High artemisinin clones can be propagated vegetatively either by cuttings or micropropagation. Further, tissue culture can be used to propagate and provide clean stock plants to disseminate for cuttings. Further studies are needed to determine if clonal propagation is cost effective. The efficiency would be a function of artemisinin content, biomass production, and costs to produce plants by cuttings or micropropagation compared to seedling propagation. The adoption of the vegetative propagation of superior genotypes, with the development of marketing channels, will provide a means to meet the growing global demand of artemisinin and its derivatives, improve human health, and lead to rural economic growth in some of the world’s poorest regions. Such value-added enterprises, filling a major void in rural health and nutrition, will reduce poverty, diversify rural incomes, and reduce gender inequity. Thus, we envision that the use of clonal propagation through *in vitro* cultures or cuttings of high-artemisinin clones of *A. annua* can become a more accessible and practical method for producing homogeneously-high artemisinin crops that can reduce the price of artemisinin-combination therapy and continue to be provide a means of generating income to Asian and African communities afflicted by poverty and malaria.

## Author Contributions

HW designed the study, devised tissue culture methods, interpreted the results, and drafted the manuscript. JP assisted in field trials and tissue culture experiments, ran the artemisinin analyses, collected and analyzed data. JF helped with study design and performed sesquiterpene analyses. JF and JJ interpreted results, contributed to the manuscript, and provided germplasm for the selection studies. TM helped design and conducted tissue culture and field studies, analyzed data, and contributed to the manuscript.

## Conflict of Interest Statement

The authors declare that the research was conducted in the absence of any commercial or financial relationships that could be construed as a potential conflict of interest.

## Footnotes

1 http://www.a2s2.org/market-data/a2s2-market-update-aug13.html
